# The Diagnosis of Dengue in Patients Presenting With Acute Febrile Illness Using Supervised Machine Learning and Impact of Seasonality

**DOI:** 10.3389/fdgth.2022.849641

**Published:** 2022-03-14

**Authors:** Damien K. Ming, Nguyen M. Tuan, Bernard Hernandez, Sorawat Sangkaew, Nguyen L. Vuong, Ho Q. Chanh, Nguyen V. V. Chau, Cameron P. Simmons, Bridget Wills, Pantelis Georgiou, Alison H. Holmes, Sophie Yacoub

**Affiliations:** ^1^Centre for Antimicrobial Optimisation, Imperial College London, London, United Kingdom; ^2^Children's Hospital 1, Ho Chi Minh City, Vietnam; ^3^Oxford University Clinical Research Unit, Centre for Tropical Medicine, Ho Chi Minh City, Vietnam; ^4^Centre for BioInspired Technology, Imperial College London, London, United Kingdom; ^5^University of Medicine and Pharmacy at Ho Chi Minh City, Ho Chi Minh City, Vietnam; ^6^Hospital for Tropical Diseases, Ho Chi Minh City, Vietnam; ^7^Institute of Vector Borne Disease, Monash University, Clayton, VIC, Australia; ^8^Centre for Tropical Medicine and Global Health, Nuffield Department of Medicine, University of Oxford, Oxford, United Kingdom

**Keywords:** dengue, supervised machine learning, diagnosis, seasonality, climate change

## Abstract

**Background:**

Symptomatic dengue infection can result in a life-threatening shock syndrome and timely diagnosis is essential. Point-of-care tests for non-structural protein 1 and IgM are used widely but performance can be limited. We developed a supervised machine learning model to predict whether patients with acute febrile illnesses had a diagnosis of dengue or other febrile illnesses (OFI). The impact of seasonality on model performance over time was examined.

**Methods:**

We analysed data from a prospective observational clinical study in Vietnam. Enrolled patients presented with an acute febrile illness of <72 h duration. A gradient boosting model (XGBoost) was used to predict final diagnosis using age, sex, haematocrit, platelet, white cell, and lymphocyte count collected on enrolment. Data was randomly split 80/20% into a training and hold-out set, respectively, with the latter not used in model development. Cross-validation and hold out set testing was used, with performance over time evaluated through a rolling window approach.

**Results:**

We included 8,100 patients recruited between 16th October 2010 and 10th December 2014. In total 2,240 (27.7%) patients were diagnosed with dengue infection. The optimised model from training data had an overall median area under the receiver operator curve (AUROC) of 0.86 (interquartile range 0.84–0.86), specificity of 0.92, sensitivity of 0.56, positive predictive value of 0.73, negative predictive value (NPV) of 0.84, and Brier score of 0.13 in predicting the final diagnosis, with similar performances in hold-out set testing (AUROC of 0.86). Model performances varied significantly over time as a function of seasonality and other factors. Incorporation of a dynamic threshold which continuously learns from recent cases resulted in a more consistent performance throughout the year (NPV >90%).

**Conclusion:**

Supervised machine learning models are able to discriminate between dengue and OFI diagnoses in patients presenting with an early undifferentiated febrile illness. These models could be of clinical utility in supporting healthcare decision-making and provide passive surveillance across dengue endemic regions. Effects of seasonality and changing disease prevalence must however be taken into account—this is of significant importance given unpredictable effects of human-induced climate change and the impact on health.

## Introduction

Dengue is an important viral infection and accounts for a considerable burden of disease worldwide. As a major cause of acute undifferentiated febrile illness (1), it is the main differential diagnosis in patients presenting with a fever during rainy season in endemic settings. Although the majority of symptomatic patients experience an uncomplicated illness, up to 5% develop severe disease associated with severe plasma leak, significant bleeding, or major organ impairment (2). Accurate diagnosis is a priority as those with dengue need to be monitored closely, and there are downstream implications on other aspects of acute febrile illnesses management such as the unnecessary of use of empirical antimicrobials as a driver of antimicrobial resistance (3).

Dengue infections exhibit a seasonal pattern and high caseloads are seen during rainy seasons as a function of vector behaviour and other factors (4). Syndromic diagnosis of dengue is widely implemented, although this process is highly dependent on training and experience (5). The use of tacit knowledge such as seasonality and reports of local outbreaks are used by clinicians to assess the overall risk of dengue—these are an important part of clinical decision-making but have not been captured in past models or guidelines. Point-of-care lateral flow assays including those which detect non-structural protein 1 and/or dengue IgM play a role in supporting diagnosis of acute dengue. Their accuracy can be variable (6) and performance affected by factors such as serotype, illness duration, and disease prevalence. Low-cost and robust molecular diagnostics are much needed and developments are underway (7), but their widespread implementation currently remains infeasible. With the backdrop of human-induced climate change, new autochthonous transmission and changing seasonal epidemiology of dengue, new tools to support clinical diagnosis, and management are warranted (8–10).

The use of a data-driven approach for disease diagnosis such as those using machine learning techniques allow for the integration of large, diverse exogenous datasets in order to empirically address pertinent clinical questions. A system capable of utilising routine healthcare data efficiently could be used to provide adjunctive support in clinical decision-making, such as in patient risk stratification (11) or disease surveillance. There is a role particularly for digital health interventions in healthcare settings with cellular connectivity but limited access to laboratory testing services, as found in many regions around the world (12). Further integration of these systems for passive real-time epidemiology could provide added-value and facilitate early outbreak detection and public health responses (13).

We developed and evaluated a supervised machine learning model using data from a prospective clinical observational study. The model takes in routine clinical and laboratory data in order to predict the probability of dengue as a final diagnosis. We subsequently examined the role of seasonality and a changing background disease prevalence, and investigated methods to maintain consistent performance throughout the year, in order to ensure that the model is of optimal clinical utility regardless of when it is utilised.

## Methods

This is a retrospective analysis of data from an observational clinical study previously published (14, 15). The aim of the work was to develop and evaluate machine learning models which could predict the final diagnosis in patients presenting with an acute febrile illness in the early phase. This study was approved by the scientific and ethical committee of the Hospital for Tropical Diseases (HTD), Ho Chi Minh City (reference 145-0420) and by the Oxford Tropical Research Ethics Committee (OxTREC, reference 146-0420) with all datasets pseudonymised prior to analyses.

### Clinical Dataset

The clinical dataset used was derived from a paediatric cohort of patients prospectively enrolled from 7 healthcare facilities located in Southern Vietnam including Ho Chi Minh City between 12th March 2010 and 10th December 2014. Children aged between 1 and 15 years old who presented within 72 h onset of an undifferentiated febrile illness consistent with dengue from the community were enrolled into the study after appropriate informed consent. Demographic, clinical information and blood samples for haematology, biochemistry, a NS1 rapid test (NS1 Ag STRIP, Bio-Rad, USA), reverse-transcriptase polymerase chain reaction (RT-PCR) were collected at time of enrolment. Ambulatory patients were followed up by daily phone calls until resolution of fever. Patients who were admitted to hospital within 6 days of enrolment were followed up in-person daily in hospital and a second blood sample was collected for paired serology.

### Dengue Outcome Definitions

A definition of laboratory-confirmed dengue was used in line with standard definitions. This was defined as compatible clinical findings with one or more of the following: (i) Positive RT-PCR, (ii) Positive NS1 result in blood, or (iii) IgM seroconversion (Panbio, Australia) for patients who attended. Patients with an acute febrile illness without any positive dengue-specific laboratory tests were classified as belonging in the “other febrile illness” (OFI) group for this study.

### Feature Selection

Feature variables were selected on the basis of available clinical data in the dataset collected during early illness and expert opinion. These features have to be sufficiently basic and available to ensure that they are available in a variety of healthcare settings to allow for widespread implementation. The final demographic and laboratory features chosen for the final models were: patient age, gender, day of illness, and full blood count results on enrolment (haematocrit, platelet, white cell, and lymphocyte count).

### Model Development

A gradient boosting machine learning algorithm, XGBoost was used (16)—this is an ensemble tree-based algorithm which accounts for missing values. There was missing data for 8 (0.001%) patients and additional imputation was not performed in this study. The entire dataset underwent an initial stratified random split into 80/20 ratio to form a training and hold-out test set, respectively, with the latter not used in model development at any stage. Using data from the training set alone, a hyperparameter grid search process with 10-fold cross validation was used to optimise model fitting, with the area under the receiver operating curve (AUROC) used as the primary scoring parameter to evaluate model performance. Optimal cut offs for the probabilistic classifier were determined using Youdens J-statistic. Isotonic regression was used for model calibration.

### Seasonality

Seasonality was explored using a rolling window model. We took a 30-day window which was progressively moved forward in time until the end of the study period. Within each window all cases of acute febrile illnesses recruited within this timeframe were included and used as the test set, evaluated against a model trained on all data not included in this particular timeframe. The window was moved forward by a 1-day increment and process repeated until the end of the study period (Supplementary Figure 1).

From this method we adopted either a constant, or dynamic threshold model. For the constant model positive classes of dengue were defined when probabilistic output for the model is >0.5. For the dynamic model we took the assumption that the qualitative and quantitative nature of acute febrile illnesses presenting close in time were more similar to those presenting further away from each other. Through adjusting the decision threshold using a continuous learning process based on the characteristics of the preceding data window, the true positive rate (sensitivity) or false positive rate (1/specificity) was adjusted with a subsequent impact on PPV and NPV values.

As the primary clinical role for the model was the safe triage of patients at low risk of dengue for community follow-up and to reduce hospital admission, as well as to prioritise clinical evaluation for those with a higher probability of dengue, we chose to fix the negative predictive value of the model to achieve a performance above 90%.

Corresponding climate data in the form of average precipitation and temperature for the country was obtained from world bank data (17). A full description of methods is attached in the Supplementary Appendix.

### Role of Funder

The funder of the study had no role in study design, data collection, data analysis, data interpretation, or writing of the article. The corresponding author had full access to all the data in the study and final responsibility for the decision to submit for publication.

## Results

### Baseline Information

We included 8,100 patients enrolled in the clinical study in our analysis. The median age was 6 years old (Interquartile range, IQR 3–9 years), 44% were female (3,541/8,100), and the median duration of fever at enrolment was 3 days (IQR 3–4 days). Overall, 27.6% (2,244/8,100) patients had a laboratory confirmed diagnosis of dengue of which 2,186 had a positive RT-PCR, 1,134 had a positive NS1 and 973 had a positive IgM and/or paired serology. There was <1% missing data for each feature included in the model.

A baseline description of the cohort, and univariate comparisons between patients with a final diagnosis of dengue and OFI are shown in Table 1. Patients with dengue were significantly older (9 vs. 5 years old), had a higher initial haematocrit (39 vs. 37%), a lower platelet count (180 vs. 243 × 10^9^ cells/L), and a lower white cell count (4.9 vs. 9.0 × 10^9^ cells/L), when compared with OFI (*p*-values all <0.001).

**Table 1:** Baseline description of the cohort by final diagnosis.

	**All patients (*n =* 8,100)**	**Dengue (*n =* 2,240)**	**OFI (*n =* 5,860)**	***P*-value**
Median age in years (IQR)	6 (3–9)	9 (6–11)	5 (3–8)	<0.01
Duration of illness (days)	3 (3–4)	4 (3–4)	3 (2–4)	<0.01
Female sex	3,541 (44%)	976 (44%)	2,565 (44%)	0.9
Haematocrit	38 (36–40%)	39 (37–41%)	37 (35–40%)	<0.01
Platelet count (cells × 10^9^/L)	227 (181–278)	180 (141–225)	243 (202–292)	<0.01
White cell count (cells × 10^9^/L)	7.7 (5.2–11.2)	4.9 (3.6–6.9)	9.0 (6.4–12.5)	<0.01
Lymphocyte count (cells × 10^9^/L)	1.8 (1.1–2.8)	1.1 (0.8–1.6)	2.1 (1.4–3.2)	<0.01

*Mann-Whitney and Chi Squared tests were used for univariate analyses between the dengue and OFI groups*.

### Model Development and Testing

We applied a stratified random split on the whole dataset to create a training development set (*n* = 6,480 patients; 27.7% dengue) and a hold-out test set (*n* = 1,620; 27.2% dengue). A calibrated XGBoost model was fitted to the training set only. This was done using grid search process looking at optimal hyperparameters in 10-fold stratified cross validation.

The median performance in cross-validation of the optimal model was as follows: AUROC of 0.86 (Interquartile range 0.84–0.86), specificity 0.92, sensitivity 0.56, positive predictive value (PPV) 0.73, and negative predictive value (NPV) 0.84. The model output used was probabilistic in nature and isotonic calibration with 10-fold cross-validation was used and the Brier score was 0.13 demonstrating good calibration.

**Figure 1:**
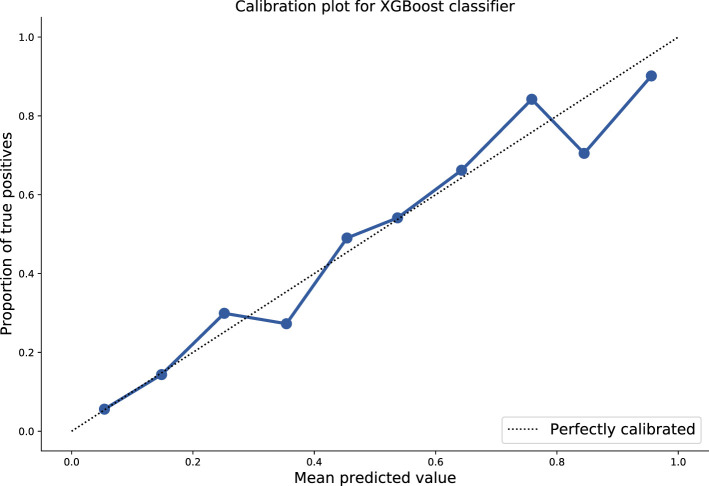
Plot of mean predicted values as function of probabilistic model output, against proportion of true positives. Ten-fold isotonic calibration was used for optimisation resulting in a Brier score of 0.13.

We then evaluated this optimised model against the independent hold-out test set tuned using the J-statistic and an AUROC of 0.86, specificity of 0.78, sensitivity of 0.79, PPV of 0.58, and NPV of 0.91 was achieved. These results and the calibration curve are shown in Table 2, Figure 1, respectively.

**Table 2:** Results of model cross validation, hold out set performance and calibration.

	**Sample size**	**Dengue diagnoses**	**AUROC**	**Specificity**	**Sensitivity**	**Positive predictive value**	**Negative predictive value**	**Brier score**
Cross validation	6,480	1,792 (27.7%)	0.86 (IQR 0.84–0.86)	0.92	0.56	0.73	0.84	0.13
Hold out set testing	1,620	441 (27.2%)	0.86	0.78	0.79	0.58	0.91	0.13

*The full dataset (n = 8,100) patients is randomly split into a 80/20 ratio with the latter hold out set not used for model training*.

### Performance of the Model Over Time and Seasonality

The prevalence of dengue changes each month and is related to transmission factors including weather and climate. During periods with higher rainfall (typically May—December in Vietnam) a higher proportion of patients in the study were diagnosed with dengue. Within our dataset we observe this seasonal pattern in the ratio of dengue diagnoses against OFI cases.

As performance of any test is affected by underlying prevalence, we explored changes in positive and negative predictive value of the model over time. A longitudinal model was constructed using rolling window cross-validation (see methods). The performances between a constant threshold baseline model and a dynamic model were then compared. In the latter, dynamic model the decision threshold for each window was iteratively tuned to achieve a negative predictive value of at least 90%. For each prediction at any timepoint within the dynamic model therefore, the optimal threshold was determined by characteristics of the cases in the preceding 30-day window.

**Figure 2:**
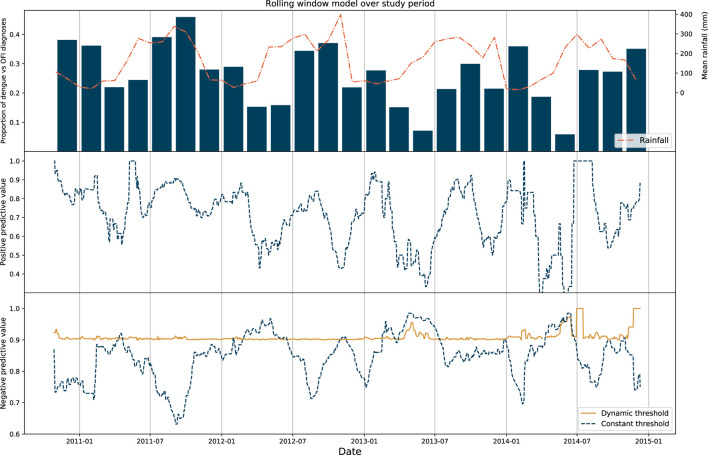
Results of rolling window model. Top: Proportion of dengue diagnoses grouped in 2-month bins plotted against mean rainfall in Vietnam for the entire study period. Middle: Positive predictive value of the rolling window model for the constant threshold model only, with each point on the graph representing the upper range of the 30-day window used as the test set. Bottom: Negative predictive value of the rolling window model for the constant and dynamic model, with the NPV of the latter model fixed to > 90%.

We show that PPV and NPV changes significantly over time in the constant model [median performance values of 0.73 (IQR 0.60–0.83) and 0.86 (IQR 0.81–0.90), respectively] and these display a degree of seasonality with respect to precipitation and dengue prevalence: in general, NPV was highest when prevalence of dengue was lowest. Consistent NPV performance was subsequently achieved using the dynamic model (median 0.90, IQR 0.90–0.91) using a changing threshold approach. These results are displayed in Figure 2.

In order to validate the thresholds determined by the dynamic model, we applied this model to generate predictions for patients admitted within a 7-day period immediately ahead of the rolling window, as a form of hold-out set testing. We show that median NPV for the entire study period was 0.91 (IQR 0.86–0.95). Sensitivity analyses were also performed for windows of 7, 14, and 21 days (Supplementary Table 1).

A secondary model with the inclusion of climatic factors (monthly rainfall and temperature from the World Bank dataset) as features into the model did not result in differences to overall predictive performance. The results are shown in the Supplementary Table 2 and implications discussed in the following section.

## Discussion

We developed supervised machine learning models trained on a large paediatric cohort of patients attending healthcare facilities in Southern Vietnam. Using basic clinical and laboratory features, the optimised model was able to discriminate between with dengue or OFI in a cohort of patients presenting with an acute febrile illness. The performance metrics of the model (AUROC 0.86) and calibration (Brier 0.13) supports a role for clinical implementation. We demonstrated that the static version of the model was associated in variable performance metrics over time, as a function of changing disease prevalence and seasonality. Application of a dynamic threshold resulted in consistency of the negative predictive value throughout the year.

Our work is placed at the convergence between the roles of informatics and diagnostics in clinical decision-making. Models which provide disease probabilities can be of utility, especially when integrated with electronic healthcare record systems. These systems offer an extra layer of safety for patient care and may assist in rapid and automatic patient triage. The latter role would be particularly important in settings where high seasonal dengue caseloads and resource limitations may lead to healthcare services being rapidly overwhelmed (18). Features used in our model (age, sex, and parameters from full blood count) are widely available and overall discrimination performance was higher compared with previously published models (15, 19). Incorporation into routine hospital laboratory systems can further support passive dengue surveillance: in turn this can inform public health interventions including local vector control efforts, preparation healthcare facility capacity and disease-specific measures (20). The accurate classification of acute febrile illnesses has direct impacts on patient clinical outcomes and supports appropriate use of empirical antimicrobials, as a means to tackle antimicrobial resistance (21). Strategies to scale up digital health interventions represents an ongoing priority of the World Health Organisation (22).

We observed changes in model performance when the data was partitioned into periods spanning 1 month. These changes were attributed to seasonality and underlying prevalence of both dengue, and the diagnoses which make up the OFI group. However, inclusion of basic climatic variables did not improve predictive performance of the model and we hypothesise that this is reflective of the complexity contained in the system. A dynamic model was developed whereby more recent cases in time continuously informed the optimal decision threshold for prediction. This model was able to maintain the negative predictive value of the model over the study period to a high (>90%) level when validated, with direct implications for patient care.

Understanding relationships between disease and climate will be increasingly crucial in the context of human-induced climate change. These changes are likely to affect disease prevalence, timings of outbreaks, geographical areas at risk, and downstream impact on population wellbeing in a significant (and unpredictable) manner (23). Although warm temperatures with high humidity are preferential for the *Aedes* mosquito vector in dengue—the interactions between climate, vector survival, reproduction, and feeding behaviour are complex (24). As an urban disease, local physical environments within pockets of densely-populated areas and human factors such as behaviour and mobility play a significant role in disease transmission.

It is therefore possible that the use of data of much greater granularity from meteorological, hydrological, entomological, and behavioural sources could translate into models with better performance. Novel methods of data acquisition utilising mHealth or drone technologies could provide the necessary substrates for more precise modelling (8). Clinical prediction models have traditionally focused only on patient variables, but machine learning approaches are inherently scalable systems and can integrate large datasets from diverse sources. The future coupling of clinical machine learning models with these diverse, non-clinical datasets including climate, vector, environmental, and behavioural data could ultimately enable greater understanding and allow for better decision support (13). However, these increasingly complex models which make use of ecological data are also likely to restrict interpretability, particularly in terms causality between data and outcomes. Being unable to understand the strength of relationships between the components could ultimately lead to a less robust model. Translation of these models with the intention of guiding decision-making should ideally address these issues in an explicit manner.

Strengths of our study include the large sample size used, balanced dataset, low proportion of missing data, and the good quality of collected data as part of a prospective clinical study. We utilised robust processes including cross-validation and independent hold-out set testing for model development which increases the validity of findings. The good degree of model calibration further supports its use as a probabilistic classifier: this is particularly important but neglected quality in models intended for use in clinical decision-making (25). A consistent negative predictive value of > 90% throughout the year in model metrics also means it is likely to translate into clinical utility when implemented. This work was done as part of a multidisciplinary collaboration (vital.oucru.org) and there are future plans and mechanisms for clinical translation and prospective model testing.

There were limitations—the dataset used was derived from a study with focus only on children in Vietnam. Specific selection biases particular to the local healthcare context and study procedures might limit translation of these findings to another setting. Nonetheless the study serves as proof of principle that basic information, when applied through a machine learning pipeline, can offer additional insights relevant to patient care. Variations in performance across time could also be subject to sampling biases as recruitment was uneven throughout the study—although this effect is limited because of the large sample sizes.

We show that variability in model performance exists across time. The inclusion of climatic data in the analyses was done with the hypothesis that environmental variables which can be measured, namely rainfall and temperature are closely associated with the prevalence of infections and has impact on model discrimination. However, we were unable to clearly define the relationship between climatic factors on model performance in this study, nor improve our existing model. We speculate this is in part because only temporally aggregated, country-wide data was used in the study whereas there is support that localised weather information is required to account for the significant spatiotemporal variations of dengue incidences in Vietnam (26).

We defined the OFI group as those without a positive dengue test on enrolment. However, the majority of patients were managed as outpatients and did not have follow up serology to definitively exclude dengue. It is therefore possible that patients with dengue were also misclassified in this group. The same OFI group was likely heterogenous in nature throughout the year (comprising of different proportions of diseases such as respiratory viral infections, vector borne diseases, bacterial infections over the year), but lack of further diagnostics data, or data describing vector/host characteristics limited further analysis in this patient group. Ideally, a multi-class model using data from individual diseases would be of benefit. Studies which aim to investigate aetiologies of febrile illnesses are currently underway, and could support development of such models in the future (27).

In conclusion we used a supervised machine learning approach to classify patients according to diagnosis in a cohort of children presenting with an early acute febrile illness. The model developed was of a standard suitable for clinical use with an AUROC of 0.86 and we adjusted for changing performance over time using a dynamic threshold. Accounting for seasonality and in-depth understanding on the complex interactions between climate, climate change, and infectious diseases through research is needed to ensure healthcare systems adapt to changing disease burdens in the future.

## Data Availability Statement

The data analysed in this study is subject to the following licences/restrictions: The dataset comprises of individual clinical trial data and any access for analysis will be subject to subsequent approvals by research ethics committees. Please contact the authors for information. Requests to access these datasets should be directed to d.ming@imperial.ac.uk.

## Author Contributions

DM and BH conceived and designed the study, extracted and processed the data, and drafted the initial manuscript. SS undertook additional data analysis and model development. NT, NC, CS, and BW were involved in the design and data collection of the original clinical study. PG, AH, and SY provided advice throughout the study. All authors reviewed the final version of the manuscript and meet criteria for authorship in the ICMJE Recommendations.

## Funding

This work was supported by the Wellcome Trust grant (215010/Z/18/Z). DM and BH receive their salaries from, and are supported by the grant. The funding source had no role in the design, data collection, analysis or writing of the manuscript.

## Author Disclaimer

AH was a National Institute for Health Research (NIHR) Senior Investigator. The views expressed in this publication are those of the author(s) and not necessarily those of the Department of Health and Social Care NHS, or the National Institute for Health Research.

This report is independent research and the views expressed are those of the author(s) and not necessarily those of the NHS, the NIHR, or the Department of Health and Social Care or Public Health England.

## Conflict of Interest

The authors declare that the research was conducted in the absence of any commercial or financial relationships that could be construed as a potential conflict of interest.

## Publisher's Note

All claims expressed in this article are solely those of the authors and do not necessarily represent those of their affiliated organizations, or those of the publisher, the editors and the reviewers. Any product that may be evaluated in this article, or claim that may be made by its manufacturer, is not guaranteed or endorsed by the publisher.
