# Supplementary material for: The Diagnosis of Dengue in Patients Presenting With Acute Febrile Illness Using Supervised Machine Learning and Impact of Seasonality
Source: Front Digit Health. 2022 Mar 14;4:849641. doi: 10.3389/fdgth.2022.849641 (PMC8963938; doi:10.3389/fdgth.2022.849641)
Supplement: Supplementary file 1 [file Data_Sheet_1.DOCX]

**Supplementary appendix**

**The diagnosis of dengue in patients presenting with acute febrile illness using supervised machine learning and impact of seasonality**

**Methods supplement**

An XGBoost classifier algorithm was used for model fitting using the following features (age, sex, haematocrit (%), platelet count, white cell count, lymphocyte count) collected on early illness presentation. As a tree-based algorithm, the data was not transformed or scaled. Missing data (<0.01%) was handled within the algorithm without imputation. The following parameter grid was employed for hyperparameter tuning, using the area under the receiver operator curve (AUROC) as the main performance metric:

     'xgb__eta': [0.01,0.05,0.075,0.1],

     'xgb__min_child_weight': [0.05,0.1,0.2],

     'xgb__max_depth': [3,4,5,6],

     'xgb__gamma': [0.01,0.1,0.15,0.2],

     'xgb__n_estimators':[25,50,75,100,150],

A stratified 10-fold cross-validation process was used on the training set (n=6480) and models were calibrated using isotonic calibration.

**Rolling windows**

Rolling window analyses were performed using a window size of 30 days. This was chosen as a timeframe sufficiently large enough to give meaningful results but close enough to be reflective of the underlying seasonality. The median number of patients for each 30-day timeframe is 161 (73-234 patients). For each window the optimal threshold for negative predictive value was determined by iterative tuning of the decision threshold until the desired NPV was attained. All predictions within that window would then utilise this optimised threshold.


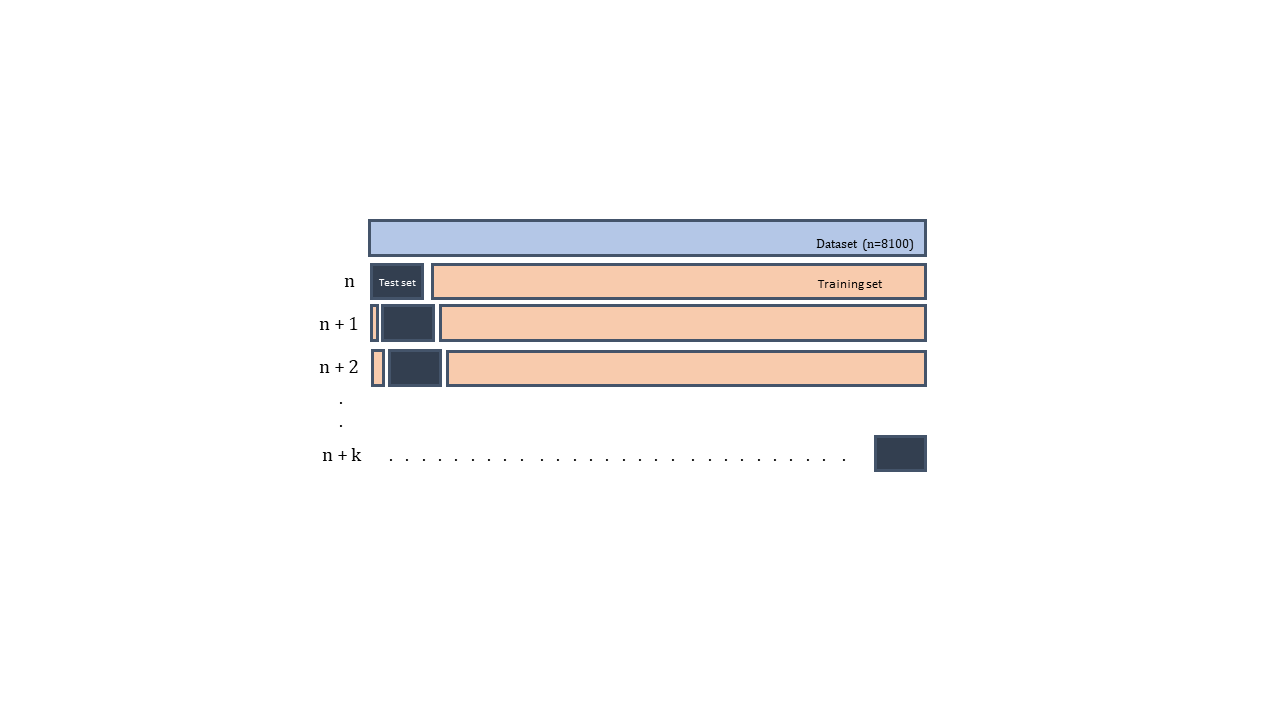


Supplementary Figure 1 – Rolling window model: the entire dataset (n=8100) was arranged according to chronological order from 2010 to 2014. A rolling window consisting of a 30 day period was initially used as the test set at timepoint n with all remaining data used as training set. The window was then incremented by 1 day and the process repeated until k, the number of unique dates between the start and end of the study.

In order to validate the utility of the optimised threshold, we verified the negative predictive value by using patient data obtained in the immediate 7-day period following the rolling window. We performed a sensitivity analysis by varying the rolling window size to 7, 14, 21 or 30 days. Results are shown below:

| Rolling window size (days) | Median NPV | Interquartile range |
| --- | --- | --- |
| 7 | 0.90 | 0.85-0.95 |
| 14 | 0.91 | 0.85-0.96 |
| 21 | 0.91 | 0.86-0.96 |
| 30 | 0.91 | 0.86-0.95 |

**Seasonality data**

Supplementary table 1 – Validation of the dynamic model and threshold adjustment showing that an overall median NPV of >0.90 is achieved through varying rolling window sizes.

We included climatic data (mean monthly rainfall and temperature from World Bank) into the basic models to explore if this reduced the variability of the model over time. We partitioned the dataset into 3-month blocks and used a leave one group out cross-validation approach whereby each block served as a test set to a model trained by the remaining data. We show that performance and distributions were not significantly different between the two models. The results are as follows:

|  | AUROC | Specificity | Sensitivity | Positive predictive value | Negative predictive value |
| --- | --- | --- | --- | --- | --- |
| Baseline model (IQR) | 0.85 (0.83-0.88) | 0.92 (0.90-0.94) | 0.56 (0.50-0.60) | 0.73 (0.69-0.79) | 0.84 (0.83-0.86) |
| Model with climatic features (IQR) | 0.86 (0.82-0.89) | 0.91 (0.88-0.96) | 0.56 (0.47-0.63) | 0.74 (0.67-0.80) | 0.84 (0.83-0.87) |

Supplementary table 2 – Performance metrics for baseline model (age, sex, full blood count) and model with climate variables (monthly rainfall and temperature) through a leave one group out approach. Figures show median values and brackets show interquartile ranges.
